# Shifts in fungal community diversity and potential function under natural forest succession and planted forest restoration in the Kunyu Mountains, East China

**DOI:** 10.1002/ece3.70055

**Published:** 2024-08-16

**Authors:** Ping Zhu, Xinyu Hu, Qiang Zou, Xiaoyan Yang, Bohan Jiang, Jincheng Zuo, Xinfu Bai, Jianqiang Song, Nan Wu, Yuping Hou

**Affiliations:** ^1^ School of Life Sciences Ludong University Yantai P.R. China; ^2^ Yantai Science and Technology Bureau Yantai Science and Technology Innovation Promotion Center Yantai P.R. China; ^3^ Department of Park Yantai Kunyu Mountain Forest Station Yantai P.R. China; ^4^ School of Resources and Environmental Engineering Ludong University Yantai P.R. China

**Keywords:** fungal community, fungal functional groups, reforestation, secondary forest succession, tree species

## Abstract

Soil fungi participate in various ecosystem processes and are important factors driving the restoration of degraded forests. However, little is known about the changes in fungal diversity and potential functions under the development of different vegetation types during natural (secondary forest succession) and anthropogenic (reforestation) forest restoration. In this study, we selected typical forest succession sequences (including *Pinus densiflora* Siebold & Zucc., pine‐broadleaf mixed forest of *P. densiflora and Quercus acutissima* Carruth., and *Q. acutissima*), as well as natural secondary deciduous broadleaved mixed forests and planted forests of *Robinia pseudoacacia* on Kunyu Mountain for analysis. We used ITS rRNA gene sequencing to characterize fungal communities and used the FUNGuild database to predict fungal functional groups. The results showed that forest succession affected fungal β‐diversity, but not the α‐diversity. There was a significant increase in Basidiomycota and a decrease in Ascomycota in the later successional stage, accompanied by an increase in the functional groups of ectomycorrhizal fungi (ECM). Conversely, planted forests exhibited decreased fungal α‐diversity and altered community compositions, characterized by fewer Basidiomycota and more Ascomycota and Mucoromycota. Planted forests led to a decrease in the relative abundances of ECM and an increase in animal pathogens. The TK content was the major factor explaining the distinction in fungal communities among the three successional stages, whereas pH, AP, and NH_4_^+^ were the major factors explaining community variations between natural and planted forests. Changes in vegetation types significantly affected the diversity and functional groups of soil fungal communities during forest succession and reforestation, providing key insights for forest ecosystem management in temperate forests.

## Graphical abstract

We studied the changes in fungal diversity and potential functions under the development of different vegetation types during natural (secondary forest succession) and anthropogenic (artificial reforestation) forest restoration. The results show that forest succession affected fungal β‐diversity, but not the α‐diversity. However, artificial reforestations were found to reduce overall soil fungal diversity and alter community composition.

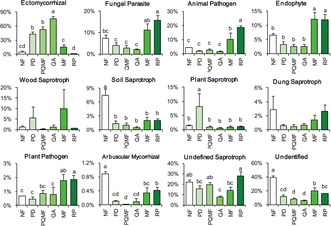

## 1 INTRODUCTION

Soil fungi play a vital role in soil nutrient cycling, are closely related to plants, and regulate plant community dynamics and productivity in forest ecosystems (Harris, 2009; Tomao et al., 2019). They are extremely diverse and functionally versatile and mainly comprise symbiotic, saprotrophic, and pathogenic fungi. Mycorrhizal fungi, such as arbuscular mycorrhizal fungi (AMF) and ectomycorrhizal fungi (ECM), are symbiotic with plant roots and provide nutrients and water for their hosts in exchange for plant carbon (Brundrett & Tedersoo, 2018). Mutualistic symbiotic interactions promote host plant growth (Franco et al., 2014; Roger et al., 2013), improve plant resistance and adaptability to stress (Begum et al., 2019), and create an underground network among plants to transport nutrients (Taylor, 2006). Saprotrophic fungi can degrade soil organic matter (SOM) and plant litter and supply soil nutrients (i.e., nitrogen and phosphorus) to support plant growth in most forest ecosystems (Talbot et al., 2013). Pathogenic fungi generally have negative interactions with plants, inhibiting plant growth and altering plant diversity and community composition (Bagchi et al., 2014). Therefore, soil fungi are of considerable importance in promoting plant growth and supporting ecological stability in forest ecosystems.

Fungal communities are susceptible to various environmental changes in climate (Martíınez‐García et al., 2017), land use (Spurgeon et al., 2013), plant species (Dassen et al., 2017; Hedĕnec et al., 2023), and soil physicochemistry (Glassman et al., 2017; Wang et al., 2019). The changes in plant species and soil environment could lead to substantial shifts in the soil fungal community. Changes in plant species during forest succession or reforestation can alter the soil microenvironment by introducing litter and root exudates (Han et al., 2021; Smith et al., 2015). During succession from coniferous forest to broad‐leaved forest, broadleaf litter has higher nitrogen and phosphorus content and a lower carbon and nitrogen ratio than coniferous litter. This can lead to a higher litter decomposition rate to facilitate the accumulation of soil‐available nutrients (Augusto et al., 2015). Therefore, the available soil nutrient content of broad‐leaved forests is sufficiently high to support fungal community growth. Changing plant species composition can result in the recruitment of species‐specific microbial groups via the production of root exudates (Santoyo, 2022). Therefore, changes in plant species composition with natural forest succession or reforestation are closely associated with changes in the diversity and community structure of soil microorganisms.

Substantial changes in soil fungal species diversity have been observed during plant succession (Ren et al., 2019; Reyes et al., 2019). However, the varied patterns can be inconsistent, which may be related to the vegetation type and soil environment (Balami et al., 2021; Zhao et al., 2023). Fungal community composition is highly sensitive to changes in successional stages (Bachelot et al., 2018). For example, a universal shift in fungal communities occurs from Ascomycota to Basidiomycota‐dominant communities during succession (Jiang et al., 2021; Yan et al., 2020). Changes in the fungal community directly affect its function, which, in turn, drives forest community succession via soil nutrient availability and plant–soil feedbacks. A high relative abundance of soil ECM in mid‐ to late‐successional stages leads to positive biotic plant–soil feedbacks and strongly promotes the performance of slow‐growing coniferous seedlings to favor successional development (Zhao et al., 2023). However, to date, most prior studies have focused on the changes in fungal diversity and community composition during vegetation succession. In this context, relatively little is known about the changes in the functional characteristics of fungi along the forest successional stages. Understanding the changes in community dynamics and potential functions of fungi during forest succession can provide more direct insights into how changes in the environment affect microbial processes related to forest ecosystem functions.

Reforestation is an important way of forest vegetation restoration. The ecological process of reforestation leads to rapid changes in the aboveground vegetation in terms of tree species, plant diversity, community composition, litter, and soil properties, which play an important role in controlling soil fungal communities. However, the effects of planted forests on soil fungal communities can vary (Carson et al., 2010; Zhang, Li, et al., 2023). For instance, there can be higher fungal diversity and production in planted *Pinus* forests than in natural *Quercus* forests (Oria‐de‐Rueda et al., 2010). Planted forests of *Eucalyptus globulus* or *Pinus pinaster* significantly alter the fungal community structure (Carson et al., 2010). Therefore, to date, our understanding of the effects of reforestation (especially planting trees) on soil fungal communities has remained relatively limited. In this context, it is necessary to investigate the responses of soil fungal communities to reforestation to promote the healthy development of forest communities and maintain forest ecosystem stability.

Kunyu Mountain, located east of the Jiaodong Peninsula, China, is the native land of *Pinus densiflora* Siebold & Zucc. and is the largest and best‐protected natural distribution center of *P. densiflora* worldwide. This area plays a vital role in water and soil resource conservation and is the most important ecological screening on China's Shandong Peninsula. The forest community mainly comprises *P. densiflora*, interspersed with mixed forests of *P. densiflora* and *Quercus acutissima* Carruth., as well as stands of *Q. acutissima*. This distribution exemplifies a typical successional sequence transitioning from coniferous to broad‐leaved forests. The area also features natural mixed forests, which include species such as pine, oak, and *Robinia pseudoacacia* L., alongside planted pure forests of *R. pseudoacacia*. The changes in plant species under forest secondary succession and reforestation may have significant effects on the soil fungal community. Therefore, we hypothesized that soil fungal community diversity and function varied during secondary forest succession and reforestation, and their variation pattern differed. In conclusion, the primary aims of this study were to (1) clarify how the diversity and composition of soil fungal communities change during forest succession and reforestation; (2) explore the changes in fungal functional groups during forest succession and reforestation; and (3) identify the key environmental factors regulating their diversity and functional shifts.

## 2 MATERIALS AND METHODS

### 2.1 Study site

The study site is located in the Kunyu Mountain National Nature Reserve (37.20° to 37.31° N, 121.62° to 121.85° E), Shandong Province, China. This area is characterized by a warm temperate monsoon climate, with an average annual temperature of 11.9°C, an average annual precipitation of 984.4 mm, and a frost‐free period of 200–220 d. The soil is classified as brown soil. Since the 1950s, this area had suffered from large‐scale damage of pine caterpillars, leading to many diseased trees were felled. Then reforestation was carried out by various methods, such as tree and shrub mixed forest, coniferous and broad‐leaved mixed forest, and 80% of the original pure pine forest was turned into mixed forest. In the middle and late 1980s, this area was closed to forest cultivation, which accelerated secondary succession forming a majority of *P. densiflora* forest, interspersed with mixed forests larch and deciduous trees. In this study, five main forest types and non‐forest (NF) land were selected. The main forest types include natural pine forests of *P. densiflora* (PD), natural pine‐broadleaf mixed forests of *P. densiflora* and *Q. acutissima* (PQMF), and natural broadleaved forests of *Q. acutissima* (QA), which constitute a natural secondary succession series, and natural secondary deciduous broadleaved mixed forests (MF), including *Sorbus alnifolia* (Sieb. et Zucc.) K. Koch, *Ailanthus altissima* (Mill.) Swingle, *Kalopanax septemlobus* (Thunb.) Koidz., etc., and planted forest of *R. pseudoacacia* (RP).

### 2.2 Soil sampling

In November 2020, five field plots (10 m × 10 m) were established within each forest type, and each plot was approximately 50 m apart. We selected five sampling points in each field plot, taking the center of the square as the sampling center, and four samples equidistant from the central sampling point were collected. Soil samples were collected from the 0 to 10 cm depth of surface soil in each field plot using a stainless‐steel collar and were mixed into one composite sample. In total, five soil samples were obtained in five plots for each forest type, thus we collected a total of 30 soil samples (5 forest types × 5 samples +1 non‐forest × 5 samples). Each composite soil sample was divided into two subsamples. One sample was immediately frozen in liquid nitrogen, transported to the laboratory as soon as possible, and stored at −80°C until DNA extraction. The other sample was placed in sterile bags and stored at −20°C for the determination of soil physicochemical properties.

### 2.3 Soil property determination

Soil pH was determined in the supernatant of 1:2.5 soil–water mixtures with a pH meter (Mettler, Switzerland). SOM content was measured following the K_2_Cr_2_O_7_‐H_2_SO_4_ oxidationreduction colorimetric method. Soil total nitrogen (TN) was determined by a Vario Micro Cube elemental analyzer (Elementar). Total phosphorus (TP) was measured by the molybdenum blue method after wet digestion with H_2_SO_4_ + HClO_4_. Total potassium (TK) was determined following digestion with HNO_3_ and HClO_4_ by a flame photometric detector. Ammonium (NH_4_^+^) and nitrate (NO_3_^−^) were extracted with 2 M KCl solution using a 1:10 soil/extractant ratio and measured using a nutrient autoanalyzer (Seal, Germany). Available phosphorus (AP) was determined via sodium bicarbonate extraction using the molybdenum blue method. Available potassium (AK) was extracted with ammonium acetate and quantified using a flame photometric detector. All the soil properties were determined according to the method described by Bao (2000).

### 2.4 DNA extraction, PCR amplification, and high‐throughput sequencing

Total genomic DNA was extracted from 0.5 g soil using a FastDNA Spin Kit (MP Biomedical, USA), following the manufacturer's instructions. A NanoDrop 1000 spectrophotometer (Thermo Fisher Scientific, USA) was used to determine the concentration of the extracted DNA. The ITS1 region of the fungal rRNA gene was amplified using the forward primer ITS1F (5′‐CTTGGTCATTTAGAGGAAGTAA‐3′) and the reverse primer ITS2R (5′‐GCTGCGTTCTTCATCGATGC‐3′). Polymerase chain reaction (PCR) was performed in triplicate using 20 μL of reaction mixture containing 2 μL buffer, 0.2 μL rTaq polymerase, 2 μL dNTPs (2.5 mM), 0.8 μL of each primer (5 μM), 10 ng of genomic DNA, and 0.2 μL of bovine serum albumin (BSA). The PCR for fungi was performed using a ABI GeneAmp 9700 (Applied Biosystems). Thermal cycling conditions were 95°C for 3 min followed by 30 cycles of 95°C for 30 s, 50°C for 30 s, and 72°C for 45 s, and a final extension at 72°C for 10 min. Paired‐end sequencing was performed using the Illumina MiSeq platform (Illumina, USA) at Mega Genomics Health Technology Co., Ltd. (Beijing, China).

### 2.5 Sequence data processing

Raw sequences were trimmed and quality‐filtered using QIIME (v.1.9.1) (Caporaso et al., 2010). Joined paired‐end reads were filtered using a series of quality standards, including a quality score higher than 20, length longer than 200 bp, no ambiguous bases, and homopolymers less than 6 bp. The remaining sequences were assigned to operational taxonomic units (OTUs) using Uparse v.11 with 97% sequence similarity (Edgar, 2013), and chimeric sequences were identified and removed. Singleton sequences were excluded from subsequent analyses. The taxonomy was assigned using the UNITE (version 8.0) fungal ITS database (Nilsson et al., 2019). We used FUNGuild (Nguyen et al., 2016) to analyze the functional groups of the fungi in the soil. Only the two levels of “Highly Probable” and “Probable” were retained for subsequent analyses. Raw data were deposited in the NCBI SRA database under the accession number PRJNA1032641.

### 2.6 Statistical analyses

One‐way analysis of variance and Tukey's HSD test were performed to determine the differences in soil properties, relative abundances of fungal taxa, and functional groups using SPSS (v20.0, SPSS Inc., USA). The α‐diversities of the fungi were calculated based on random resampling of 39,600 reads per sample, including the Shannon, Simpson, ACE, and Chao indices. Nonmetric multidimensional scaling (NMDS) was performed to evaluate the variations in soil fungal β‐diversity based on the Bray–Curtis dissimilarity matrices at different successional stages and between natural and planted forests. Analysis of similarity (ANOSIM) was conducted to examine significant differences in the fungal community composition (Clarke, 1993). A distance‐based redundancy analysis (db‐RDA) was performed to examine the influence of environmental variables on fungal communities and functional groups. Spearman's correlation analysis was performed to explore the associations between α‐diversity, or relative abundance of fungi, and soil physicochemical characteristics. NMDS, ANOSIM, db‐RDA, and heatmap plots were constructed using the *vegan* and *pheatmap* packages in R (version 4.0.2). Fungal co‐occurrence networks were structured based on Spearman's correlation matrix from the OTU tables. We visualized fungal co‐occurrence networks using Gephi (v 0.9.0; Mendes et al., 2014) and analyzed the topological parameters, including node number, edge number, average degree clustering coefficient, and network density.

## 3 RESULTS

### 3.1 Soil properties

**FIGURE 1:**
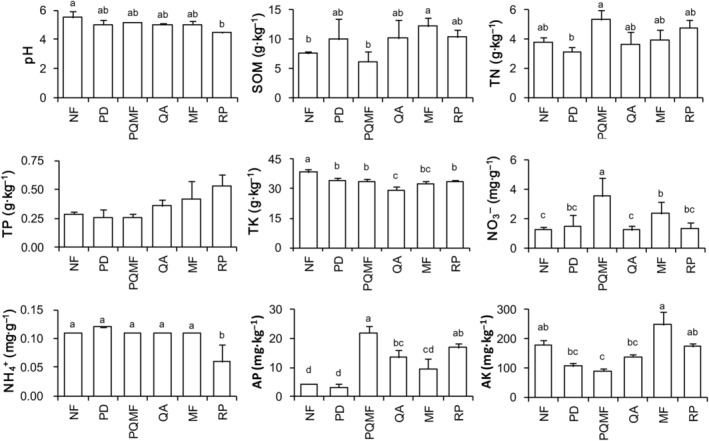
Comparison of soil properties among different forest types on Kunyu Mountain. Lowercase letters indicate statistically significant differences. MF, deciduous broadleaved mixed forest; NF, non‐forested land; PD, *P. densiflora*; PQMF, pine‐broadleaf mixed forest of *P. densiflora* and *Q. acutissima*; QA, *Q. acutissima*; RP, *R. pseudoacacia*.

Forest vegetation succession significantly affected the contents of TN, TK, NO_3_^−^, and AP, whereas pH, SOM, TP, NH_4_^+^, and AK showed no significant changes. NO_3_^−^ and AP contents increased significantly and then decreased during forest succession (Figure 1), reaching the highest levels in the PQMF during the middle period of succession. The TN content in PD of the early successional stage was significantly lower than that in PQMF of the middle successional stage, whereas the TK content was significantly lower in QA of the later successional stage (*p* < .05). Comparing soil properties among non‐forest, mixed forests, and *R. pseudoacacia* forests, the pH was the highest in the non‐forest land and significantly higher than that in *R. pseudoacacia* forest soil. The contents of SOM and NO_3_^−^ in the mixed forest soil were significantly higher than those in the non‐forest soil but were not significantly different from those in the *R. pseudoacacia* soil. The TK content in the non‐forest soil was significantly higher than that in the mixed forests and *R. pseudoacacia* forest soils. The NH_4_^+^ content in the *R. pseudoacacia* forest soil was significantly lower than that in the non‐forest and mixed forest soils, but the AP content was significantly higher.

### 3.2 Fungal diversity and community composition

**FIGURE 2:**
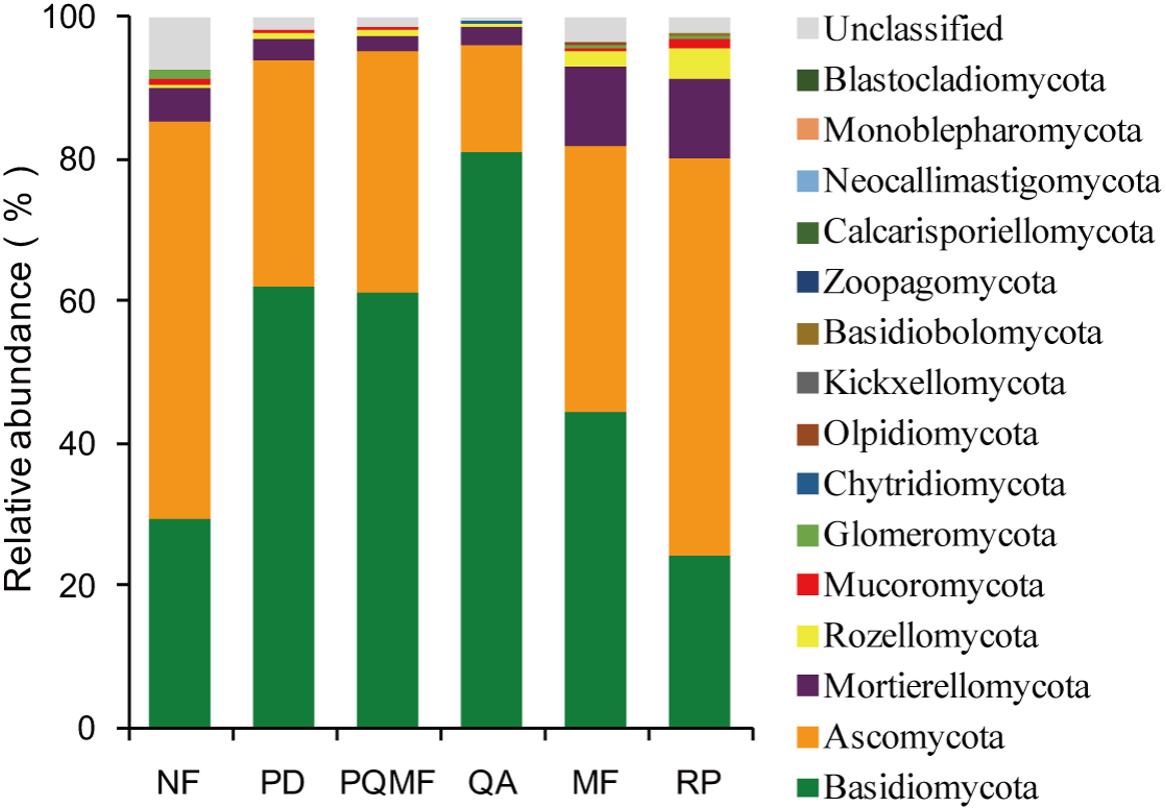
Differences in the community composition of soil fungi in different forest types at the phylum level. MF, deciduous broadleaved mixed forest; NF, non‐forested land; PD, *P. densiflora*; PQMF, pine‐broadleaf mixed forest of *P. densiflora* and *Q. acutissima*; QA, *Q. acutissima*; RP, *R. pseudoacacia*.

The sequencing results showed that 1,979,113 optimized sequences were obtained after quality control with an average sequence length of 248 bp, and 3862 OTUs were obtained by clustering. The fungal sequences were primarily represented by Basidiomycota (50.0%, mainly Agaricomycetes and Tremellomycetes), Ascomycota (38.28%), and Mortierellomycota (5.86%) (Figure 2). Other taxa such as Rozellomycota and Mucoromycota were minor (<2%). At the genera level, the fungal sequences were maily represented by *Russula* (15.09%), *Saitozyma* (6.80%), *Penicillium* (5.80%), and *Mortierella* (5.71%).

**FIGURE 3:**
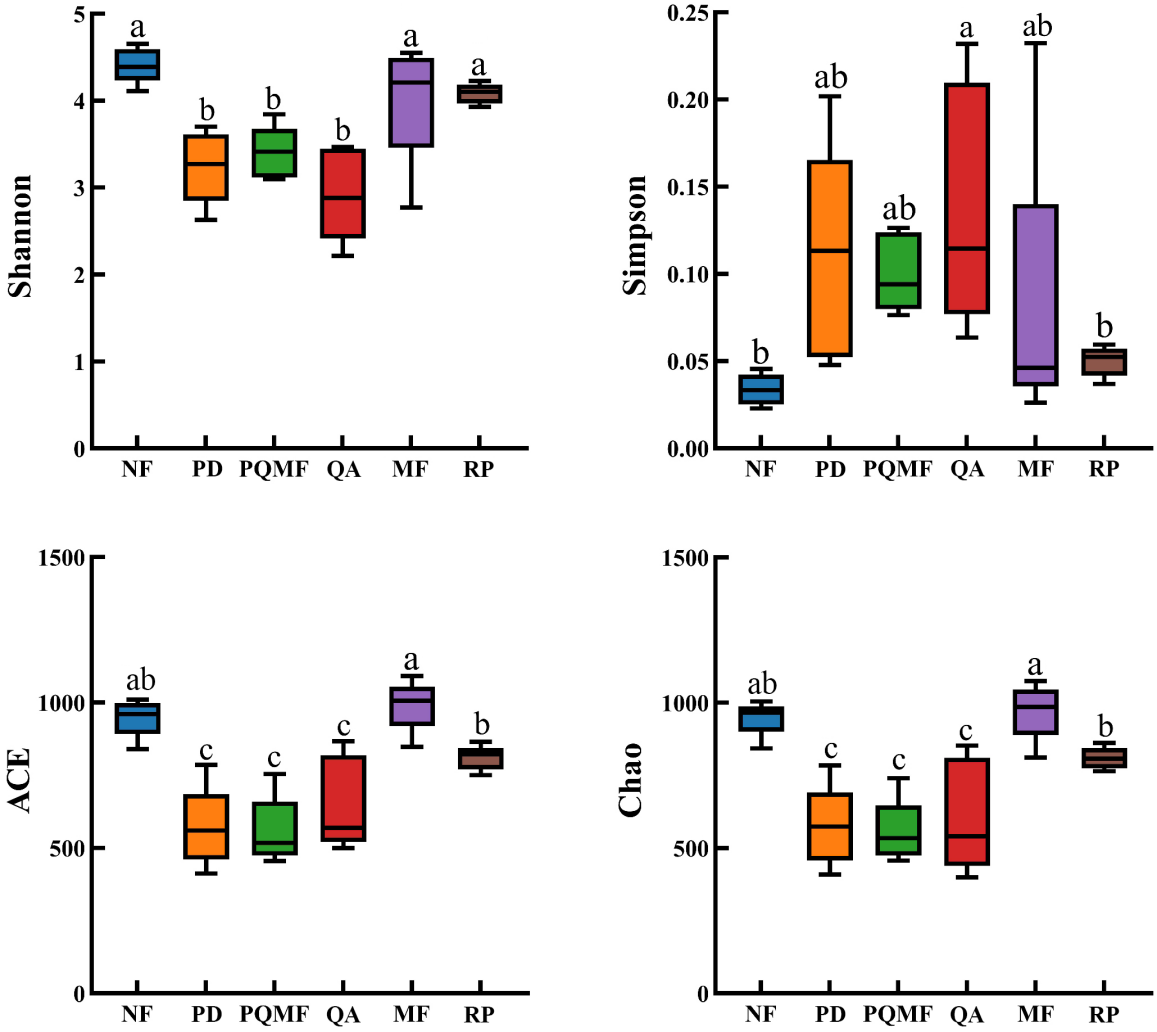
Variation in soil fungi α‐diversity among three forest successional stages and between natural and planted forests. Lowercase letters indicate statistically significant differences. MF, deciduous broadleaved mixed forest; NF, non‐forested land; PD, *P. densiflora*; PQMF, pine‐broadleaf mixed forest of *P. densiflora* and *Q. acutissima*; QA, *Q. acutissima*; RP, *R. pseudoacacia*.

The α‐diversity index of fungi did not change significantly across different succession stages, but significantly changed between mixed forests and *R. pseudoacacia* forests (Figure 3). The Shannon, ACE, and Chao indices of non‐forest, mixed forests, and *R. pseudoacacia* forests were significantly higher than those in three successional stage forests. The ACE and Chao indices in the mixed forests were significantly higher than those in the *R. pseudoacacia* forest.

**FIGURE 4:**
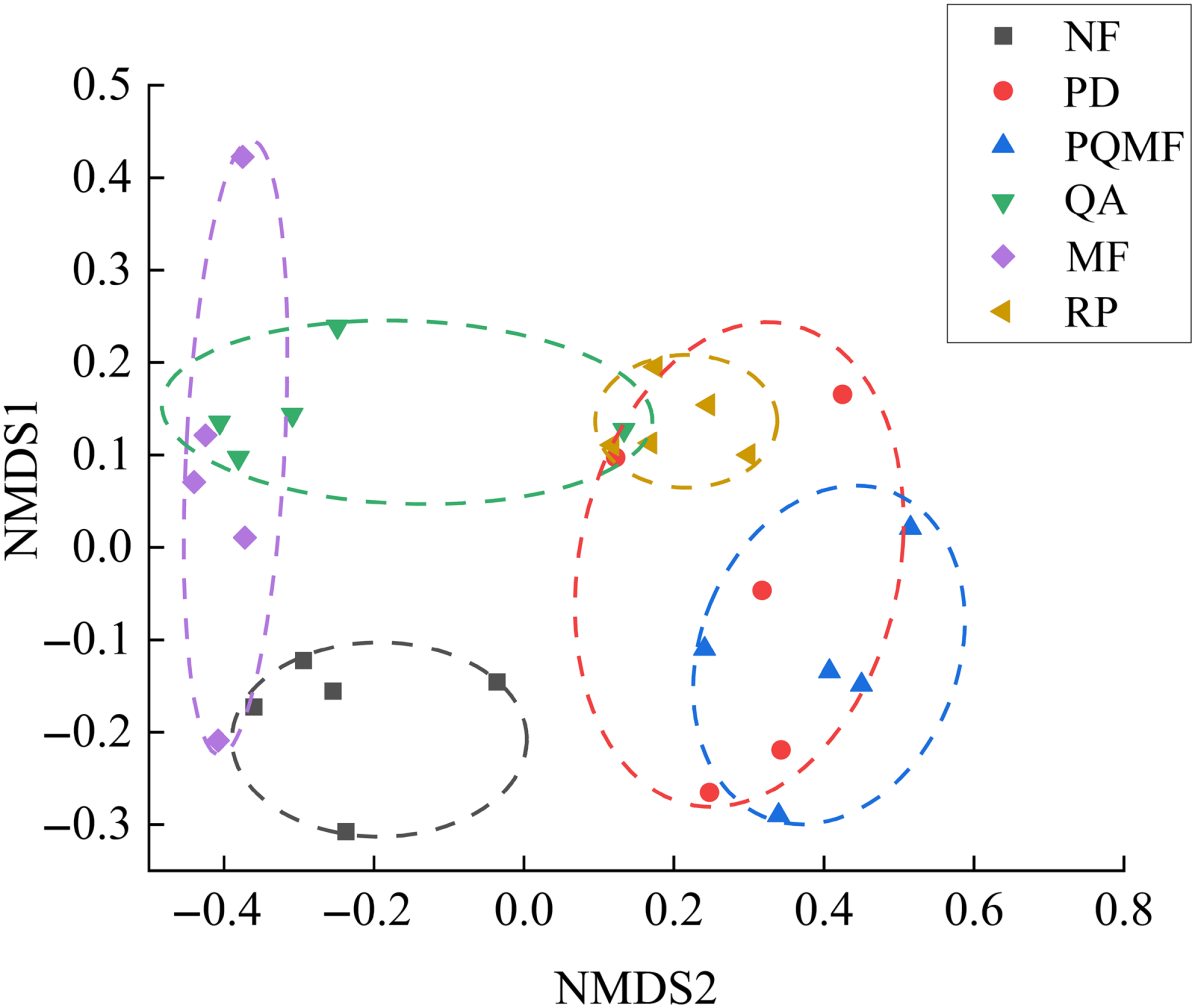
Plots of nonmetric multidimensional scaling (NMDS) based on the Bray–Curtis distance of samples for the fungal communities at three successional stages and in natural and planted forests. MF, deciduous broadleaved mixed forest; NF, non‐forested land; PD, *P. densiflora*; PQMF, pine‐broadleaf mixed forest of *P. densiflora* and *Q. acutissima*; QA, *Q. acutissima*; RP, *R. pseudoacacia*.

**FIGURE 5:**
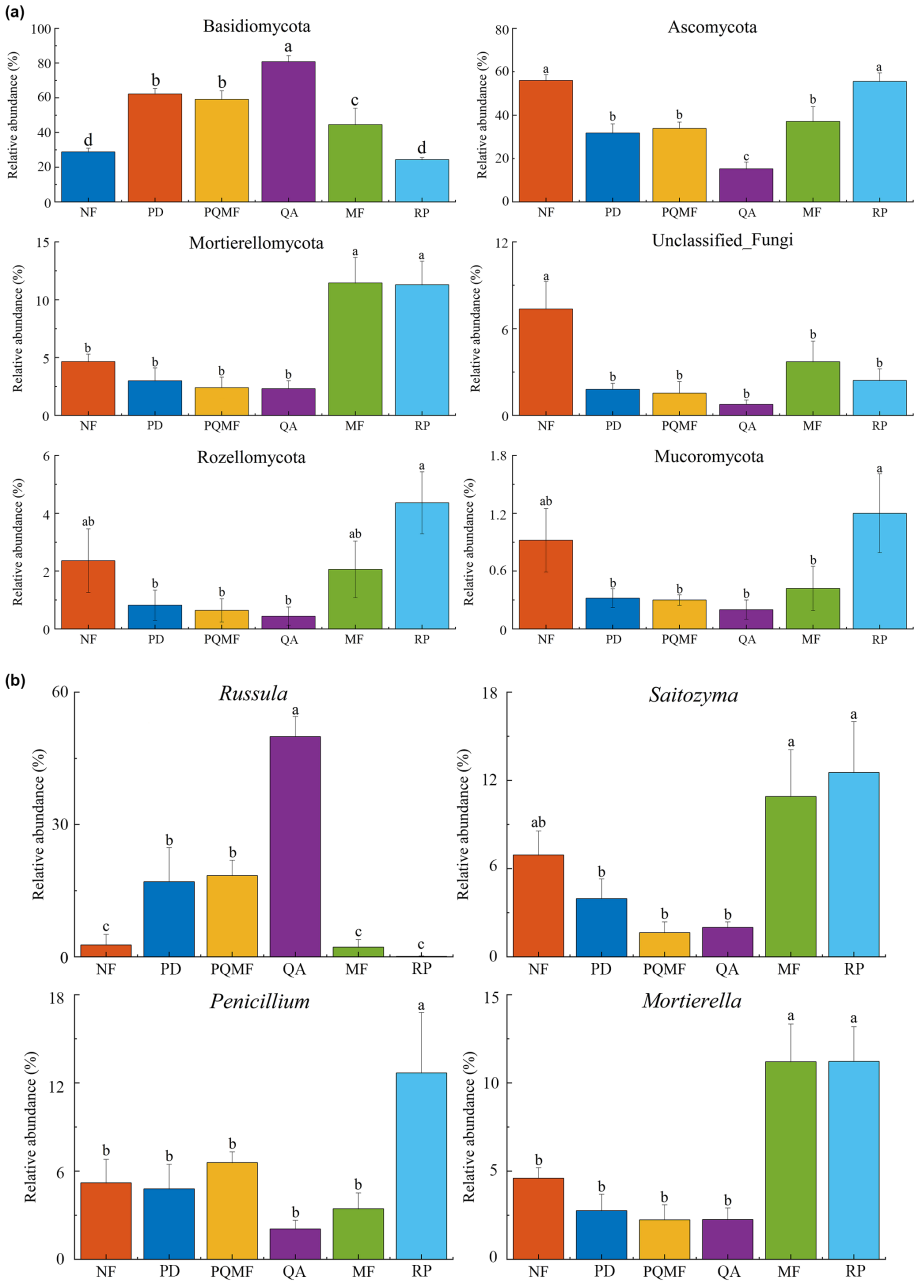
Comparisons of the relative abundances of the major taxa of fungi in different forest types at the phylum (a) and genus (b) levels. Lowercase letters indicate statistically significant differences. MF, deciduous broadleaved mixed forest; NF, non‐forested land; PD, *P. densiflora*; PQMF, pine‐broadleaf mixed forest of *P. densiflora* and *Q. acutissima*; QA, *Q. acutissima*; RP, *R. pseudoacacia*.

The fungal β‐diversity varied significantly among the three succession stages (*p* < .022) and between natural and planted forests (*p* = .039) (Figure 4 and Table 1). We further analyzed the major groups that produced differences in the fungal community structure of different forest types. Among the three succession stages, only the relative abundances of Basidiomycota and Ascomycota showed significant differences at the phylum level, whereas those of Mortierellomycota, Rozellomycota, and Mucoromycota showed no evident changes (Figure 5a). The relative abundance of Basidiomycota was significantly higher in the later successional stage QA (80.85%) soil than in the early successional stage PD (62.19%) and the middle successional stage PQMF (61.15%) soils (Table S1). Meanwhile, Ascomycetes showed the opposite pattern and were significantly lower in the QA (15.31%) than in the PD (31.82%) and PQMF (33.87%) soils. At the genus level, the relative abundance of *Russula* increased significantly in the QA soil during the late succession stage (Figure 5b). Between the natural mixed forest and planted *R. pseudoacacia* forest, Basidiomycota was significantly higher in MF (44.53%) than in RP (24.39%), whereas Ascomycota (37.11% vs. 55.57% in MF and RP, respectively) and Mucoromycota (0.40% vs. 1.20% in MF and RP, respectively) decreased significantly (Table S2). At the genus level, only *Penicillium* was significantly more abundant in the *R. pseudoacacia* forest than in the mixed forest (Figure 5b).

**TABLE 1:** Difference in fungal community structure among three successional stages and between natural and planted forests based on analysis of similarity with Bray–Curtis distance.

Group	Bray–Curtis	
	*R*	*p*
*Successional stages*		
PD versus PQMF	.556	**.004**
PD versus QA	.568	**.007**
PQMF versus QA	.416	**.022**
*Natural versus planted forest*		
NF versus MF	.664	**.004**
NF versus RP	1.000	**.004**
MF versus RP	.184	**.039**

*Note*: Bold indicates *p* < .05.

Abbreviations: MF, mixed forest; NF, non‐forested land; PD, *P. densiflora*; PQMF, mixed forest of *P. densiflora* and *Q. acutissima*; QA, *Q. acutissima*; RP, *R. pseudoacacia*.

### 3.3 Co‐occurrence network complexity of fungi

**FIGURE 6:**
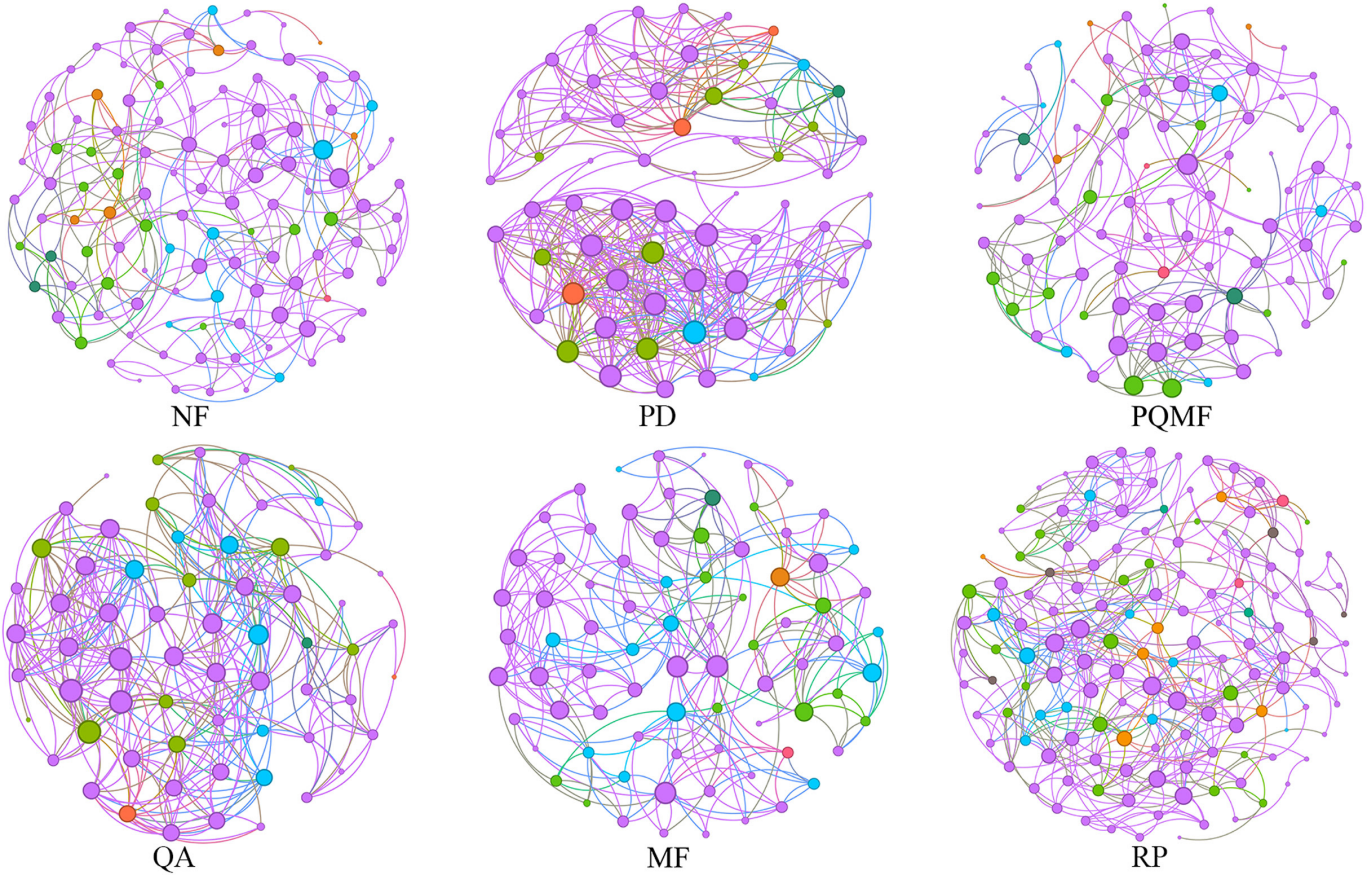
Co‐occurrence network analysis for soil fungi. MF, deciduous broadleaved mixed forest; NF, non‐forested land; PD, *P. densiflora*; PQMF, pine‐broadleaf mixed forest of *P. densiflora* and *Q. acutissima*; QA, *Q. acutissima*; RP, *R. pseudoacacia*.

Co‐occurring networks of soil fungi were constructed at the OTU level to explore the effects of forest vegetation on fungal interactions (Figure 6 and Table 2). We found that the co‐occurring networks of soil fungi differed with changes in the succession stage. These topological indices of the network, that is, average degree, density, and average clustering coefficient, showed a difference in the soil fungal community among the three forest succession types (Table 2). The number of nodes in the network first increased and then decreased with forest succession, reaching its highest value of 92 in PQMF in the middle stage of succession. Meanwhile, the number of edges, average degree, density, and average clustering coefficient first decreased in the middle stage of succession (PQMF) and then increased in the later stage of succession (QA). This indicates that the network complexity was lower in the middle stage of succession.

**TABLE 2:** The topological features of the fungal co‐occurrence networks.

Network parameter	NF	PD	PQMF	QA	MF	RP
Node number	66	66	92	65	79	142
Edge number	401	401	242	338	265	473
Modularity	0.446	0.446	0.71	0.471	0.681	0.708
Average degree	12.152	12.152	5.261	10.4	6.709	6.662
Average weighted degree	0.258	0.258	0.109	0.22	0.137	0.141
Density	0.187	0.187	0.058	0.163	0.086	0.047
Clustering coefficient	0.744	0.744	0.522	0.646	0.654	0.439
Average path length	4.048	4.048	4.085	3.185	4.303	4.429

Abbreviations: MF, mixed forest; NF, non‐forested land; PD, *P. densiflora*; PQMF, mixed forest of *P. densiflora* and *Q. acutissima*; QA, *Q. acutissima*; RP, *R. pseudoacacia*.

Between mixed forests and *R. pseudoacacia* forests, the nodes and edges of RP were higher than those of MF, but the average degree, density, and average clustering coefficient were lower. However, the average degree, density, and average clustering coefficient of both were lower than those of non‐forest.

### 3.4 Functional groups of soil fungal community

**FIGURE 7:**
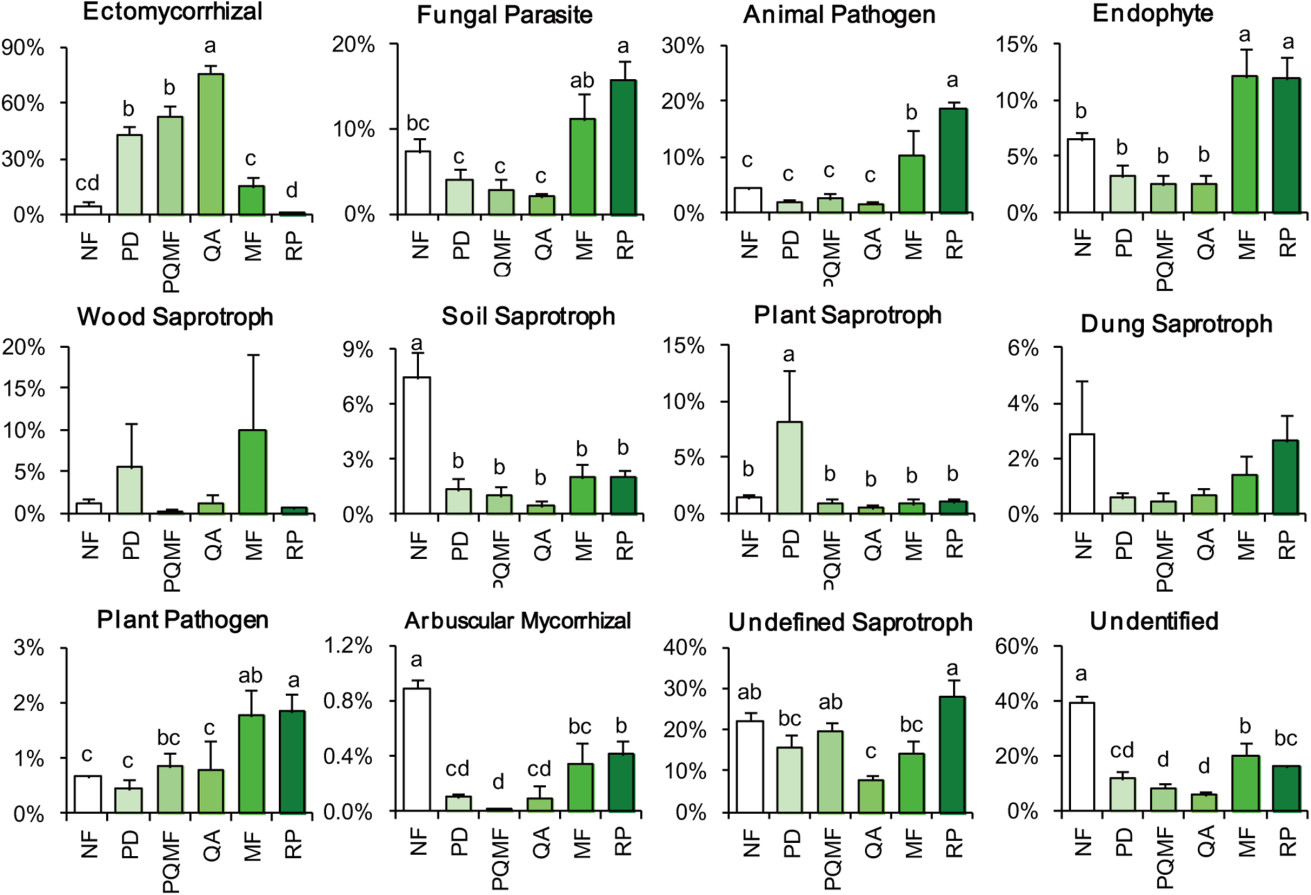
Changes in soil fungal functional groups among different stages of succession and between natural and planted forests. Lowercase letters indicate statistically significant differences.

The trophic modes of soil fungi in the different forest types were obtained using FUNGuild (Figure 7). The relative abundances of ectomycorrhizals, plant saprotrophs, and undefined saprotrophs varied significantly in forest soils at different successional stages. The relative abundance of ECM in the later successional stage QA was significantly higher than that in the early successional stage PD and the middle successional stage PQMF. In contrast, the number of saprotrophs significantly decreased in the early and middle successional stages. The relative abundances of animal pathogens, fungal parasites, endophytes, and plant pathogens were significantly higher in mixed forests and *R. pseudoacacia* forests than in non‐forests. However, soil saprotrophs and AMF were significantly lower in mixed forests and *R. pseudoacacia* forests than in non‐forests. Between mixed forests and *R. pseudoacacia* forests, ectomycorrhiza in MF was significantly higher than that in RP, but animal pathogens and undefined saprotrophs were significantly lower.

### 3.5 Correlations between fungal community and soil properties

**FIGURE 8:**
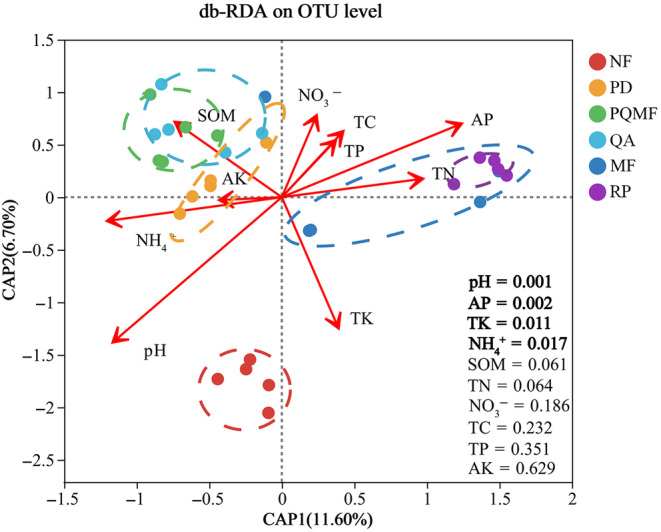
db‐RDA plots for fungal assemblages showing the covering relationship between soil properties and community structure. MF, deciduous broadleaved mixed forest; NF, non‐forested land; PD, *P. densiflora*; PQMF, pine‐broadleaf mixed forest of *P. densiflora* and *Q. acutissima*; QA, *Q. acutissima*; RP, *R. pseudoacacia*.

Db‐RDA showed that the fungal community structure of all 30 forest soil samples varied significantly with pH, TK, AP, and NH_4_^+^ (Figure 8). TK content may be the major factor explaining the distinction in fungal communities among the three successional stages, whereas pH, AP, and NH_4_^+^ may be the major factors explaining community variations between natural and planted forests.

**FIGURE 9:**
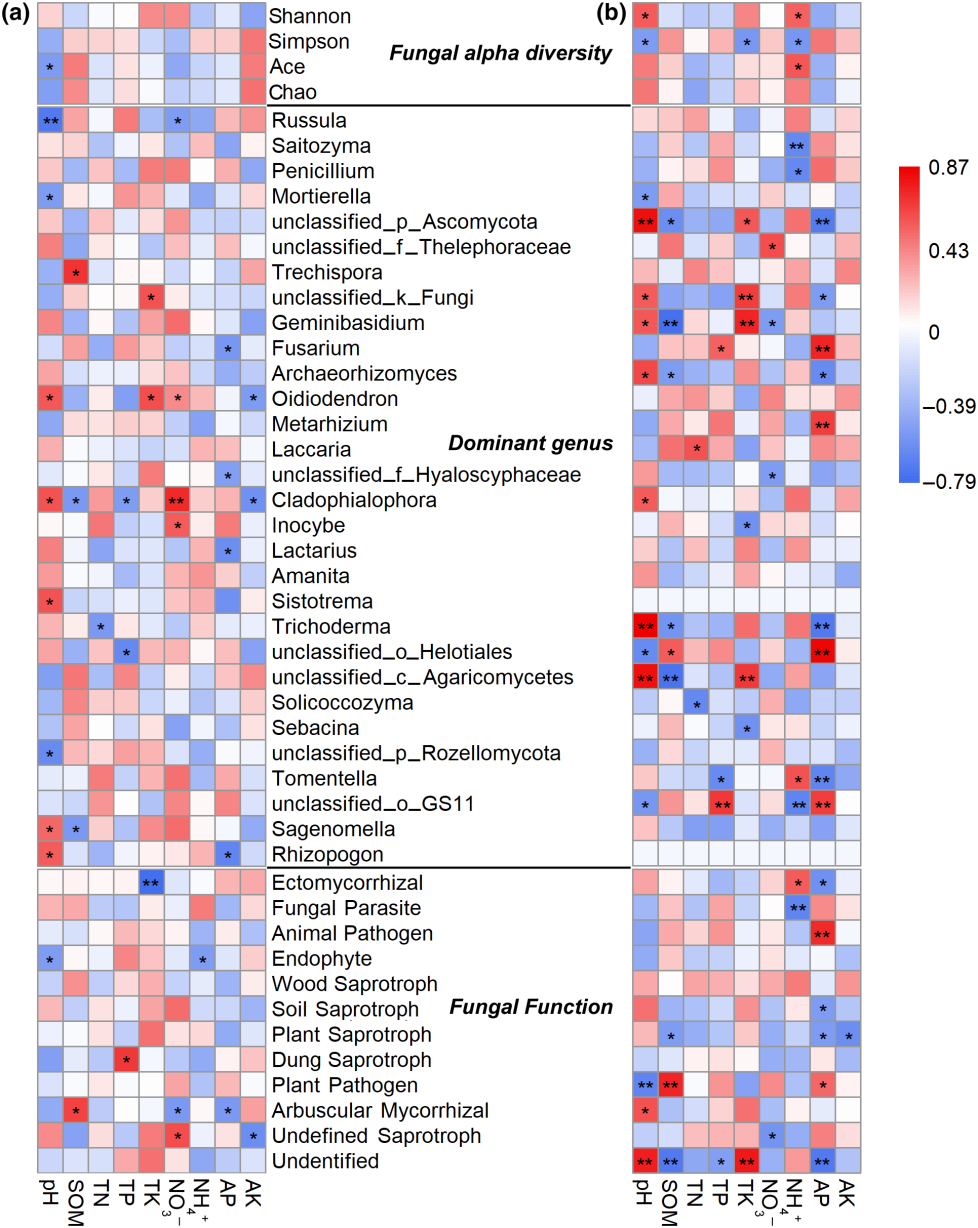
Spearman's correlations between soil properties and fungal α diversity, dominant genera, and functional groups at three successional stages (a) and in natural and planted forests (b). Asterisks and double asterisks indicate *p* values <.05 and <.01, respectively. The values of the correlation coefficients are indicated according to the color bar.

Spearman's correlation analysis was performed separately in the three successional stages and in mixed forest and *R. pseudoacacia* forests to examine the relationship between soil properties and α‐diversity, relative abundance of fungal taxa, and function (Figure 9). During the successional stages, only the ACE index was negatively correlated with pH (*p* = .045; Figure 9a). The dominant genera *Russula* (*p* = .003) and *Mortierella* (*p* = .043) were both negatively correlated with pH. The functional group Ectomycorrhizal was negatively correlated with TK (*p* < .001), and undefined saprotroph was positively correlated with NO_3_^−^ (*p* = .017) and negatively correlated with AK (*p* = .018). In non‐forest, mixed forest, and *R. pseudoacacia* forest soil samples, the Shannon index had a positive correlation with pH (*p* = .032) and NH_4_^+^ (*p* = .037), and ACE also had a positive correlation with NH_4_^+^ (*p* = .023), whereas Simpson had a negative correlation with pH (*p* = .042), TK (*p* = .029), and NH_4_^+^ (*p* = .033; Figure 9b). *Saitozyma* (*p* = .010) and *Penicillium* (*p* = .010) were negatively correlated with NH_4_^+^, while *Mortierella* was negatively correlated with pH (*p* = .048). Functionally, ectomycorrhizal was positively correlated with NH_4_^+^ (*p* = .027) and negatively correlated with AP (*p* = .019), while animal pathogens were positively correlated with AP (*p* = .002). The plant pathogen was negatively correlated with pH (*p* = .008), but positively correlated with SOM (*p* = .002) and AP (*p* = .040).

## 4 DISCUSSION

### 4.1 Different responses of α‐ and β‐diversities to forest type

Forest type significantly affected soil fungal diversity in this study, but α‐ and β‐diversity showed different patterns. Forest succession did not significantly affect the α‐diversity of fungi. Soil fungal α‐diversity has been shown to be significantly affected by edaphic variables in forest ecosystems, especially soil pH and nutrient content (Vasco‐Palacios et al., 2020; Wang et al., 2015). In this study, nonsignificant changes in fungal α‐diversity in the three succession stages may be related to there being relatively little change in soil properties, namely, pH, SOM, TP, NH_4_^+^, and AK (*p* > .05). The results of the correlation analysis also showed no significant influence of soil physicochemical properties on fungal α‐diversity during forest succession (Figure 9a). However, between natural mixed forest and planted forest, fungal α‐diversity showed significant differences. Mixed forest (MF) and non‐forest land (NF) had higher α‐diversity than *R. pseudoacacia* forest (RP). Plant species diversity in mixed forests can positively affect fungal diversity via various root exudates, nutrients, and water uptake (Nakayama et al., 2019). Therefore, diverse fungi can coexist in mixed forest ecosystems (Liu et al., 2019). Planted forests may further affect fungal diversity by changing soil physicochemical properties such as pH, TN, NH_4_^+^, AP, and AK. The correlation analysis results also indicated that soil properties were significantly correlated with α‐diversity (Figure 9b).

The soil fungal β‐diversity showed significant changes both during the forest succession stages and between natural mixed forest and planted forest of *R. pseudoacacia* (RP) (*p* < .05). This is consistent with the finding of Jiang et al. (2021) for vegetation succession in boreal forests. Different plant species select for specific soil microbial communities (Zhang, Liu, et al., 2023). This is likely because plants secrete different root exudates, that is, amino acids, organic carbon, polysaccharides, and leteolin, during their growth, which can promote or restrict microbial communities (Lombardi et al., 2018). Li et al. (2022) found that *Populus* secretes strigol and phenolic compounds, which are key drivers of bacterial and fungal communities in the rhizosphere. These findings indicate that changes in the dominant plant species have a strong influence on the fungal community.

Moreover, the changes of environmental factors also significantly affected the community structure of fungi. The RDA plot showed that TK, pH, AP, and NH_4_^+^ were important driving factors affecting the community structure of soil fungi in different forest types. However, it is worth noting that only a small fraction (about 18%) of variation in fungal community structure was explained by the selected environmental variable. In fact, besides the environmental factors, biotic interactions are known as a major control on microbial diversity (Zhou & Ning, 2017), such as predation, competition, allelopathy, parasitism, and mutualism. Li et al. (2023) found that the bacterial diversity indices and community structure were much more accurately explained by a number of eukaryotic characteristics than by the measured environmental variables and/or spatial variables alone. Over 70% of total variation in bacterial community structure could be explained by three categories of biotic interactions: parasitism, fungi–bacterial competition, and trophic structure and bacterivory. However, these biotic factors were not involved in this study, and it is necessary to further consider the effects of biotic interactions on fungal communities in the future.

### 4.2 Specific changes in the fungal community

We further analyzed the changes in fungi at different taxonomic levels. At the phylum level, Basidiomycota and Ascomycota were the most dominant phyla in all forest soils. This is consistent with other forest soils that typically contain two main and ubiquitous fungal groups (Dong et al., 2021; Štursová et al., 2020). They can degrade a fraction of plant residues and promote soil carbon accumulation (Clemmensen et al., 2013). However, in the present study, Basidiomycota and Ascomycota showed different patterns in forest succession. The relative abundance of Basidiomycota increased significantly in the late successional stage (QA) compared with that in the early (PD) and middle successional stages (PQMF), whereas Ascomycota decreased significantly. Basidiomycetes produce lignin‐degrading enzymes to decompose lignin and other recalcitrant organic matter (Alfaro et al., 2014). The refractory organic matter content in the litter composition increases with succession, contributing to an increase in Basidiomycota (Jiang et al., 2021; Yan et al., 2020). In Basidiomycota, the differences were mainly in the *Russula* genus, which was significantly higher in the late succession stage than in the early and middle succession stages. *Russula* were identified as key ECM fungi that can establish a large mycelial network to participate in nutrient cycling and energy metabolism in host plants (Twieg et al., 2007). Twieg et al. (2007) found that *Russula* had a high relative abundance in *Q. acutissima* forests, a typical tree species associated with ECM fungi. This may explain the higher *Russula* in QA of the late succession period in our study. Ascomycota are able to decompose cellulose and hemicellulose in the litter (Weber et al., 2011). In this study, the relative abundance of Ascomycota in the later successional stage (QA) was significantly lower than that in the early successional stage (PD) and the middle successional stage (PQMF). Similar results were found in studies on boreal forests in China, where Ascomycota abundance was significantly lower in QA than in mixed forests (*Q. acutissima* and *R. pseudoacacia*) in November‐sampled soils (Dong et al., 2021). However, Dong et al. (2021) found that the variation in Ascomycota was inconsistent between samples collected in May and November, indicating that the relative abundance of Ascomycota may be closely related to seasonal variation. Our study has certain limitations, particularly the fact that sample collection was confined to a single season. To gain a more nuanced understanding, future research should incorporate multiseasonal or long‐term sampling to further explore the dynamic changes in fungal taxa during forest succession.

When comparing mixed forest and *R. pseudoacacia* forest, we found that Basidiomycota decreased in RP, while Ascomycota and Mucoromycota increased. Ascomycota are generally abundant in environments with high disturbance or strong human intervention compared to virgin forest soils (Chen et al., 2017). In Ascomycota, the main differences occurred in the *Penicillium* genus, which is a highly competitive species because it can outcompete other microbes by producing large numbers of asexual spores and surviving in disturbed environments (Torres‐Cruz et al., 2018). This may account for the relatively high abundance of *Penicillium* in the planted forest soil used in the present study. *Penicillium* is typically a phosphate‐solubilizing fungus, and many strains can solubilize forms of phosphate that are unavailable to plants (Coutinho et al., 2012; Efthymiou et al., 2018). The higher relative abundance of *Penicillium* in the planted forest RF may explain the significant increase in soil AP content (Figure 1). Owing to the low bioavailability of phosphorus, a shortage of AP in soils often becomes a key factor restricting plant growth and development (Vitousek et al., 2010). Therefore, the enrichment of *Penicillium* in *R. pseudoacacia* forests is beneficial for promoting the availability of soil nutrients, which may play an important role in maintaining *R. pseudoacacia* forest growth and health.

### 4.3 Functional changes in the soil fungal community

We found that ECM increased in the late succession stages (QA). The main reason for this phenomenon may be that the QA forest in the late succession stage is a typical tree species associated with ECM fungi, forming a large mycelial network with ECM fungi that participate in nutrient cycling (Tedersoo et al., 2010). In contrast, soil nutrient contents decreased significantly in the late succession period, such as TK, NO_3_^−^, and AP. This may provide an oligotrophic environment favorable for the colonization of ECM fungi (Khalid et al., 2021). ECM fungi can promote water and nutrient absorption in host plants through the mycelial network, enhance the stress tolerance of host plants, and facilitate community succession (Kałucka & Jagodziński, 2016). In contrast to ECM fungi, the relative abundance of saprotrophs decreased with succession. Saprotrophic fungi can accelerate the decomposition of SOM to facilitate nutrient cycling in terrestrial ecosystems, among which plant saprotrophs are mainly involved in plant decomposition (Bello et al., 2021). Increasing soil carbon sources can stimulate saprotroph fungal growth, which may result in an increase in the relative abundance of saprotrophs (Marañón‐Jiménez et al., 2021). In this study, the increase in the relative abundance of plant saprotrophs in the early successional stage may be attributed to the higher carbon content of the leaf litter in coniferous *P. densiflora* forest (PD) than in broad‐leaved *Q. acutissima* forest (QA) (Lu et al., 2022). This indicates that plant species play an important role in regulating the function of soil fungal communities during forest succession.

The relative abundance of animal pathogens and undefined saprotrophs was higher in *R. pseudoacacia* forest soil than in mixed forest soil, whereas ectomycorrhizal abundance showed the opposite trend. This significant change in fungal function may be attributed to the elimination of major habitats for plant symbionts, such as ECM, following the removal of the forest floor (Tedersoo et al., 2003). ECM and saprobic fungi are most sensitive to logging disturbances (Hartmann et al., 2012). In addition, *R. pseudoacacia* is symbiotic with AMF (Yang et al., 2015). Arbuscular mycorrhizal trees have a higher relative abundance of saprotrophs and pathogens (Eagar et al., 2022; Fang et al., 2020). More attention should be paid to changes in pathogenic fungi under planted forest management.

## 5 CONCLUSION

Our study demonstrates that the development of different forest types via natural and planted restoration processes exerts a profound impact on soil fungal diversity, community composition, and functional group dynamics. Forest succession was observed to affect the fungal community composition, yet it did not alter the α‐diversity. In this typical succession sequence from coniferous *P. densiflora* to broad‐leaved *Q. acutissima* forests, a notable increase in ECM and a decrease in saprophytic fungi were evident during the later successional stages. Conversely, reforestations were found to reduce overall soil fungal diversity and alter community composition. In terms of functional groups, *R. pseudoacacia* forests decreased the relative abundance of ECM and increased the number of animal pathogens. It is worth exploring the role of specific differences in fungal taxa in forest succession and reforestations in future research.

## AUTHOR CONTRIBUTIONS

**Ping Zhu:** Methodology (equal); software (equal); writing – original draft (lead); writing – review and editing (lead). **Xinyu Hu:** Investigation (lead); methodology (equal); software (equal); writing – original draft (supporting). **Qiang Zou:** Supervision (equal). **Xiaoyan Yang:** Investigation (supporting); resources (supporting). **Bohan Jiang:** Investigation (supporting); methodology (supporting). **Jincheng Zuo:** Funding acquisition (equal); investigation (equal); methodology (equal). **Xinfu Bai:** Conceptualization (supporting). **Jianqiang Song:** Investigation (supporting); methodology (supporting). **Nan Wu:** Supervision (equal). **Yuping Hou:** Funding acquisition (equal); project administration (lead); writing – review and editing (equal).

## CONFLICT OF INTEREST STATEMENT

The authors declare that they have no conflicts of interest.

## Supporting information


Table S1.

Table S2.

## Data Availability

Raw data of fungal sequences were deposited in the NCBI SRA database under the accession number PRJNA1032641.
