# Supplementary material for: Shifts in fungal community diversity and potential function under natural forest succession and planted forest restoration in the Kunyu Mountains, East China
Source: Ecol Evol. 2024 Aug 16;14(8):e70055. doi: 10.1002/ece3.70055 (PMC11327613; doi:10.1002/ece3.70055)
Supplement: Supplementary file 1 — Table S1. [file ECE3-14-e70055-s001.docx]

**Table S1.** Comparisons of relative abundances (mean ± SE in %) of fungal taxa at phylum level among different forest types in Kunyu Mountain

| Taxonomy | NF | PD | PQMF | QA | MF | RP |
| --- | --- | --- | --- | --- | --- | --- |
| Basidiomycota | 29.28 ± 2.37d | 62.19 ± 3.20b | 61.15 ± 4.66b | 80.85 ± 3.45a | 44.53 ± 9.50c | 24.39 ± 1.16d |
| Ascomycota | 56.05 ± 2.67a | 31.82 ± 4.13b | 33.87 ± 2.94b | 15.31 ± 3.12c | 37.11 ± 6.79b | 55.57 ± 3.85a |
| Mortierellomycota | 4.66 ± 0.62b | 2.97 ± 1.11b | 2.39 ± 0.91b | 2.33 ± 0.66b | 11.46 ± 2.20a | 11.29 ± 2.02a |
| unclassified | 7.37 ± 1.91a | 1.80 ± 0.41 b | 1.55 ± 0.80b | 0.75 ± 0.32b | 3.71 ± 1.42b | 2.44 ± 0.79b |
| Rozellomycota | 0.54 ± 0.23ab | 0.78 ± 0.54b | 0.67 ± 0.40b | 0.43 ± 0.31b | 2.05 ± 0.97ab | 4.37 ± 1.08a |
| Mucoromycota | 0.92 ± 0.33ab | 0.30 ± 0.10b | 0.30 ± 0.04b | 0.18 ± 0.11b | 0.40 ± 0.23b | 1.20 ± 0.41a |
| Glomeromycota | 0.89 ± 0.06a | 0.10 ± 0.02cd | 0.01 ± 0.00d | 0.10 ± 0.09cd | 0.35 ± 0.15bc | 0.41 ± 0.10b |
| Chytridiomycota | 0.09 ± 0.02abc | 0.02 ± 0.01c | 0.03 ± 0.01bc | 0.02 ± 0.01c | 0.13 ± 0.04ab | 0.18 ± 0.07a |
| Olpidiomycota | 0.00 ± 0.00b | 0.00 ± 0.00b | 0.02 ± 0.01b | 0.01 ± 0.00b | 0.17 ± 0.12a | 0.03 ± 0.01b |
| Kickxellomycota | 0.15 ± 0.02a | 0.01 ± 0.00b | 0.00 ± 0.00b | 0.00 ± 0.00b | 0.03 ± 0.02b | 0.02 ± 0.01b |
| Basidiobolomycota | 0.01 ± 0.01b | 0.00 ± 0.00b | 0.00 ± 0.00b | 0.00 ± 0.00b | 0.00 ± 0.00b | 0.06 ± 0.03a |
| Zoopagomycota | 0.01 ± 0.01 | 0.00 ± 0.00 | 0.01 ± 0.01 | 0.00 ±0.00 | 0.00 ± 0.00 | 0.02 ± 0.01 |
| Calcarisporiellomycota | 0.02 ± 0.01a | 0.00 ± 0.00c | 0.01 ± 0.00bc | 0.00 ± 0.00c | 0.00 ± 0.00c | 0.01 ± 0.00ab |
| Neocallimastigomycota | 0.00 ±0.00 | 0.00 ± 0.00 | 0.00 ± 0.00 | 0.00 ± 0.00 | 0.03 ± 0.03 | 0.00 ± 0.00 |
| Monoblepharomycota | 0.00 ±0.00b | 0.00 ± 0.00b | 0.00 ± 0.00b | 0.00 ±0.00b | 0.01 ± 0.00b | 0.01 ± 0.00a |
| Blastocladiomycota | 0.00 ±0.00 | 0.00 ± 0.00 | 0.00 ± 0.00 | 0.00 ±0.00 | 0.00 ± 0.00 | 0.00 ± 0.00 |
