# Supplementary material for: Shifts in fungal community diversity and potential function under natural forest succession and planted forest restoration in the Kunyu Mountains, East China
Source: Ecol Evol. 2024 Aug 16;14(8):e70055. doi: 10.1002/ece3.70055 (PMC11327613; doi:10.1002/ece3.70055)
Supplement: Supplementary file 2 — Table S2. [file ECE3-14-e70055-s002.docx]

**Table S2** Comparisons of relative abundances (mean ± SE in %) of fungal taxa at genus level among different forest types in Kunyu Mountain

| Taxonomy | NF | PD | PQMF | QA | MF | RP |
| --- | --- | --- | --- | --- | --- | --- |
| Archaeorhizomyces | 7.37 ± 0.01a | 1.25±0.01b | 0.95±0.01b | 0.39±0.00b | 1.56±0.01b | 1.19±0.00b |
| Devriesia | 0.27 ± 0.00 | 0.38±0.00 | 0.63±0.00 | 0.51±0.00 | 0.72±0.00 | 0.74±0.00 |
| Cladophialophora | 2.09 ± 0.01ab | 1.89±0.00ab | 2.83±0.00a | 1.08±0.00b | 1.59±0.00b | 1.07±0.00b |
| Penicillium | 5.23 ± 0.02b | 4.82±0.02b | 6.57±0.01b | 2.06±0.01b | 3.44±0.01b | 12.69±0.04a |
| Sagenomella | 0.30 ± 0.00b | 1.00±0.00ab | 1.80±0.00a | 0.42±0.00b | 0.36±0.00b | 0.34±0.00b |
| Oidiodendron | 0.09 ± 0.00c | 4.30±0.01b | 7.28±0.02a | 0.59±0.00c | 0.15±0.00c | 0.05±0.00c |
| Pleuroascus | 0.45 ± 0.00 | 0.37±0.00 | 0.00±0.00 | 0.53±0.00 | 1.09±0.01 | 1.15±0.01 |
| Metarhizium | 0.78 ± 0.00bc | 0.24±0.00c | 0.32±0.00c | 0.45±0.00c | 3.67±0.02b | 6.76±0.01a |
| Paecilomyces | 0.18 ± 0.00c | 0.07±0.00c | 0.06±0.00c | 0.12±0.00c | 0.64±0.00b | 2.01±0.00a |
| Trichoderma | 2.52 ± 0.00a | 0.69±0.00bc | 0.86±0.00bc | 1.26±0.00b | 0.74±0.00bc | 0.36±0.00c |
| Fusarium | 1.18 ± 0.00bc | 0.65±0.00c | 0.04±0.00c | 0.34±0.00c | 3.07±0.02b | 7.54±0.01a |
| Trichocladium | 0.00 ± 0.00b | 0.00±0.00b | 0.00±0.00b | 0.01±0.00b | 0.80±0.01b | 2.82±0.00a |
| Amanita | 0.13 ± 0.00 | 0.66±0.01 | 5.93±0.04 | 0.55±0.00 | 0.00±0.00 | 0.01±0.00 |
| Laccaria | 0.00 ± 0.00 | 0.00±0.00 | 6.82±0.06 | 4.70±0.02 | 0.10±0.00 | 0.19±0.00 |
| Inocybe | 0.02 ± 0.00 | 0.00±0.00 | 7.61±0.06 | 0.39±0.00 | 1.89±0.01 | 0.00±0.00 |
| Tylospora | 0.10 ±0.00b | 1.24±0.00a | 1.54±0.01a | 0.37±0.00b | 0.08±0.00b | 0.00±0.00b |
| Rhizopogon | 0.00 ± 0.00b | 4.15±0.02a | 0.00±0.00b | 0.00±0.00b | 0.00±0.00b | 0.00±0.00b |
| Sistotrema | 0.00 ± 0.00 | 6.98±0.05 | 0.03±0.00 | 0.08±0.00 | 0.00±0.00 | 0.00±0.00 |
| Lactarius | 0.20 ± 0.00b | 5.48±0.02a | 0.14±0.00b | 3.82±0.03ab | 0.00±0.00b | 0.00±0.00b |
| Russula | 2.70 ± 0.02c | 17.21±0.08b | 18.44±0.03b | 49.90±0.05a | 2.16±0.02c | 0.12±0.00c |
| Sebacina | 0.00 ± 0.00 | 0.98±0.01 | 0.18±0.00 | 1.34±0.01 | 2.60±0.02 | 0.00±0.00 |
| Tomentella | 0.32 ± 0.00 | 0.11±0.00 | 0.88±0.00 | 0.26±0.00 | 2.83±0.02 | 0.00±0.00 |
| Trechispora | 0.93 ± 0.00 | 5.42±0.05 | 0.23±0.00 | 1.17±0.01 | 9.58±0.09 | 0.32±0.00 |
| Geminibasidium | 2.89 ± 0.00ab | 4.21±0.01a | 4.20±0.01a | 0.59±0.00bc | 0.15±0.00c | 0.81±0.00bc |
| Solicoccozyma | 1.15 ± 0.00b | 0.22±0.00c | 0.01±0.00c | 0.31±0.00bc | 2.41±0.00a | 2.03±0.00a |
| Saitozyma | 6.96 ± 0.02 | 3.97±0.01 | 1.64±0.01 | 2.00±0.00 | 10.90±0.03 | 15.34±0.02a |
| Mortierella | 4.62 ± 0.01 | 2.76±0.01 | 2.22±0.01 | 2.27±0.01 | 11.22±0.02 | 11.20±0.02 |

Lowercase letters indicate statistically significant differences.
